# Advantages offered by the double magnetic loops versus the conventional single ones

**DOI:** 10.1371/journal.pone.0211626

**Published:** 2019-02-12

**Authors:** Ferran Mocholí Belenguer, Antonio Mocholí Salcedo, Antonio Guill Ibañez, Victor Milián Sánchez

**Affiliations:** 1 Traffic Control Systems Group, ITACA Institute, Universitat Politècnica de València, Valencia, Spain; 2 Department of Electronic Engineering, ITACA Institute, Universitat Politècnica de València, Valencia, Spain; 3 Chemical and Nuclear Engineering Department, Institute of Industrial, Radiological and Environmental Safety, Universitat Politècnica de València, Valencia, Spain; Technion Israel Institute of Technology, ISRAEL

## Abstract

Due to their simplicity and operating mode, magnetic loops are one of the most used traffic sensors in Intelligent Transportation Systems (ITS). However, at this moment, their potential is not being fully exploited, as neither the speed nor the length of the vehicles can be surely ascertained with the use of a single magnetic loop. In this way, nowadays the vast majority of them are only being used to measure traffic flow and count vehicles on urban and interurban roads. This is the reason why we presented in a previous paper the double magnetic loop, capable of improving the features and functionalities of the conventional single loop without increasing the cost or introducing additional complexity. In that paper, it was introduced their design and peculiarities, how to calculate their magnetic field and three different methods to calculate their inductance. Therefore, with the purpose of improving the existing infrastructure and providing it with greater potential and reliability, this paper will focus on justifying and demonstrating the advantages offered by these double loops versus the conventional ones. This will involve analyzing the magnetic profiles generated by the passage of vehicles over double loops and comparing them with those already known. Moreover, it will be shown how the vehicle speed, the traffic direction and many other data can be obtained more easily and with less margin of error by using these new inductance signatures.

## Introduction

When trying to respond to basic questions such as the number and type of vehicles that circulate on the road [1–5], the speed at which they circulate [6–10] or the direction in which they do it [11], there is a large number of interesting studies related to different ITS technologies that can be consulted. However, despite the fact that infrastructure have changed significantly in recent years due to the continuous evolution of the technology, magnetic loops continue to be the reference traffic sensor. Proof of this is that today loop detectors still dominate the traffic installations and they are even part of the newest algorithms for traffic management in cities [12–14]. Moreover, they have proved to be very cost effective and truly complete sensors, since aside from its main application for vehicle’s classification which includes buses, trucks, cars, motorcycles and even bicycles [15–17], magnetic loops are also used for vehicle’s speed measurements [18–24], for wheels detection [25,26], for bi-directional communication between vehicles and infrastructures [27] and for vehicle’s re-identification [28].

The operation of these sensors is straightforward, since it is based on the impedance variation that is recorded in the magnetic loops during the passage of vehicles over them [29]. These are buried in the pavement and are placed in such a way that they form an oscillating circuit together with the electronic unit located in the control booth. In this manner, when a vehicle or any object built with conductive material passes through the magnetic field generated by them, there is a decrease in the global magnetic field because of the currents induced in the vehicle, which also produce a decrease in the inductance of the loop since it is proportional to the magnetic flux.

**Fig 1:**
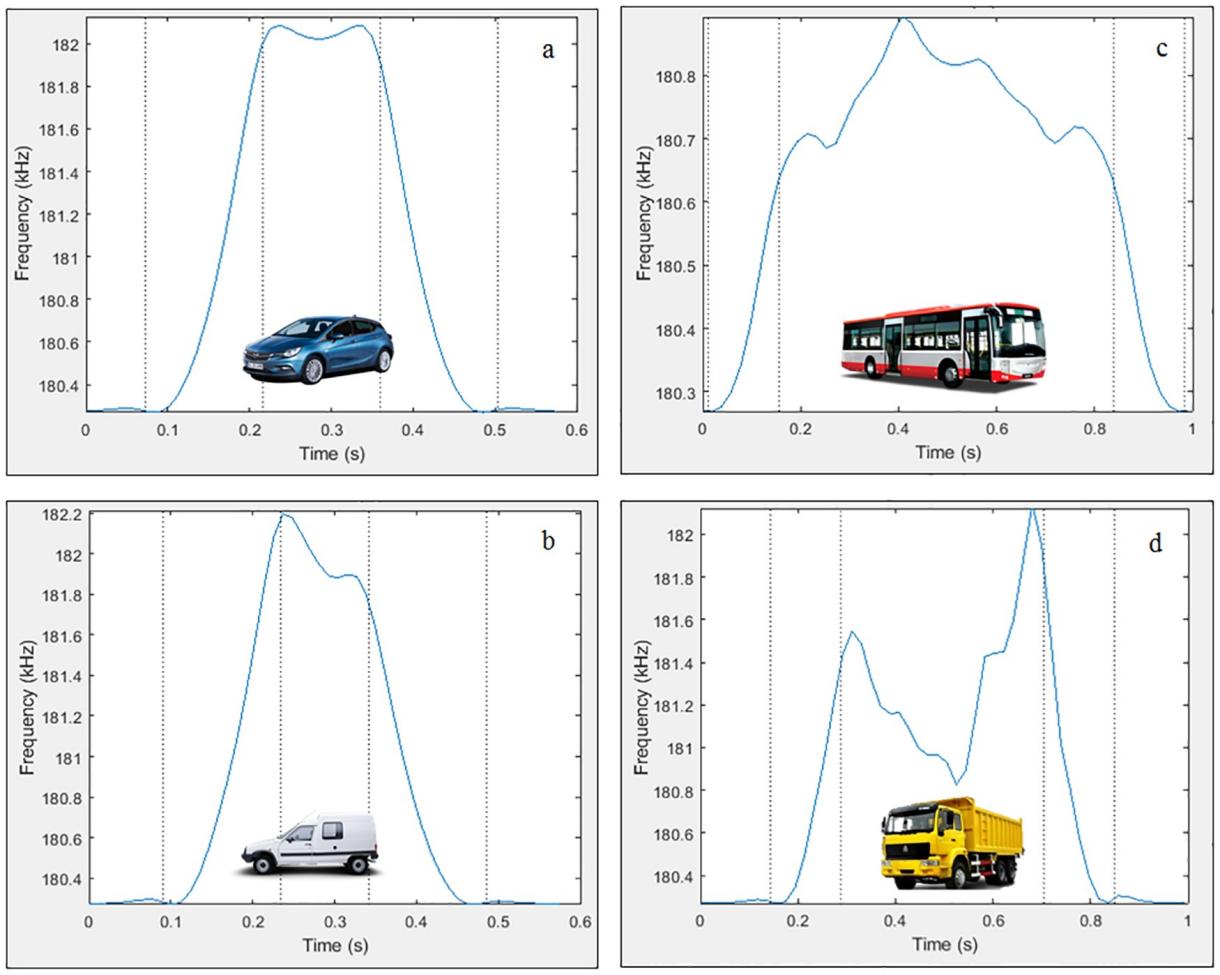
Magnetic profiles. (a) Car. (b) Van. (c) Bus. (d) Truck.

Like any resonant circuit [30], the oscillation frequency of the whole system is given by

$$f=\frac{k}{L}$$ (1)

where *k* is a constant that depends on the characteristics of the electronic components used in the construction of the oscillator circuit [30] and *L* is the loop inductance expressed in Henrys [29]. Thus, when a vehicle passes over a loop, it is obtained its magnetic profile by analyzing the inductance or frequency variation recorded. This one depends mainly on parameters related to the vehicle such as length, engine position or number of axles and is different for each type of vehicle, which allows to classify them as bicycles, motorcycles, cars, trucks and buses. This can be seen in Fig 1, where several magnetic profiles from single loops are shown. However, while these waveforms for the single loops are widely known, the magnetic profiles recorded by the passage of vehicles over double loops have not yet been studied.

Currently, speed and length estimation with only a single loop is possible by using the magnetic profile derivative on a rising or falling edge or by using specific equipment and algorithms designed for that purpose [31]. Nevertheless, these calculations are usually complex and unreliable due to their large error margins. This is the reason why in order to calculate the speed of a vehicle, its length or its direction of traffic more accurately, there are usually two single loops per lane. In this sense, the passage of a vehicle over the first loop is recorded in the detector, and after a short interval of time, the vehicle passes again over the second loop where it is also recorded. Then, as the distance between both loops is known by design, the vehicle speed, the direction of traffic and the vehicle length can be finally estimated. However, it should be noted that the classification of vehicles is not entirely reliable, since it is made according to an estimate of vehicle length and not according to its magnetic profile.

With the implementation and use of the double loops in urban and interurban roads, this problem would be solved and it would only be necessary to use a double loop in order to calculate all the previous data, since they have a simpler, more compact and more economical electronics [29]. In this way, it would be possible to develop a more reliable and lower cost traffic sensor than its predecessor and with many more features by using the same technology and operating mode already widely known. Moreover, working with a signal instead of two would facilitate the implementation of the measurement system.

Therefore, our work will aim to present and describe the main characteristics of the new magnetic profiles, the parameters that can be easily extracted from them and the advantages offered over the conventional single loops. For that purpose, we will first show the methods that are currently being used to calculate these parameters.

### Vehicle speed

**Fig 2:**
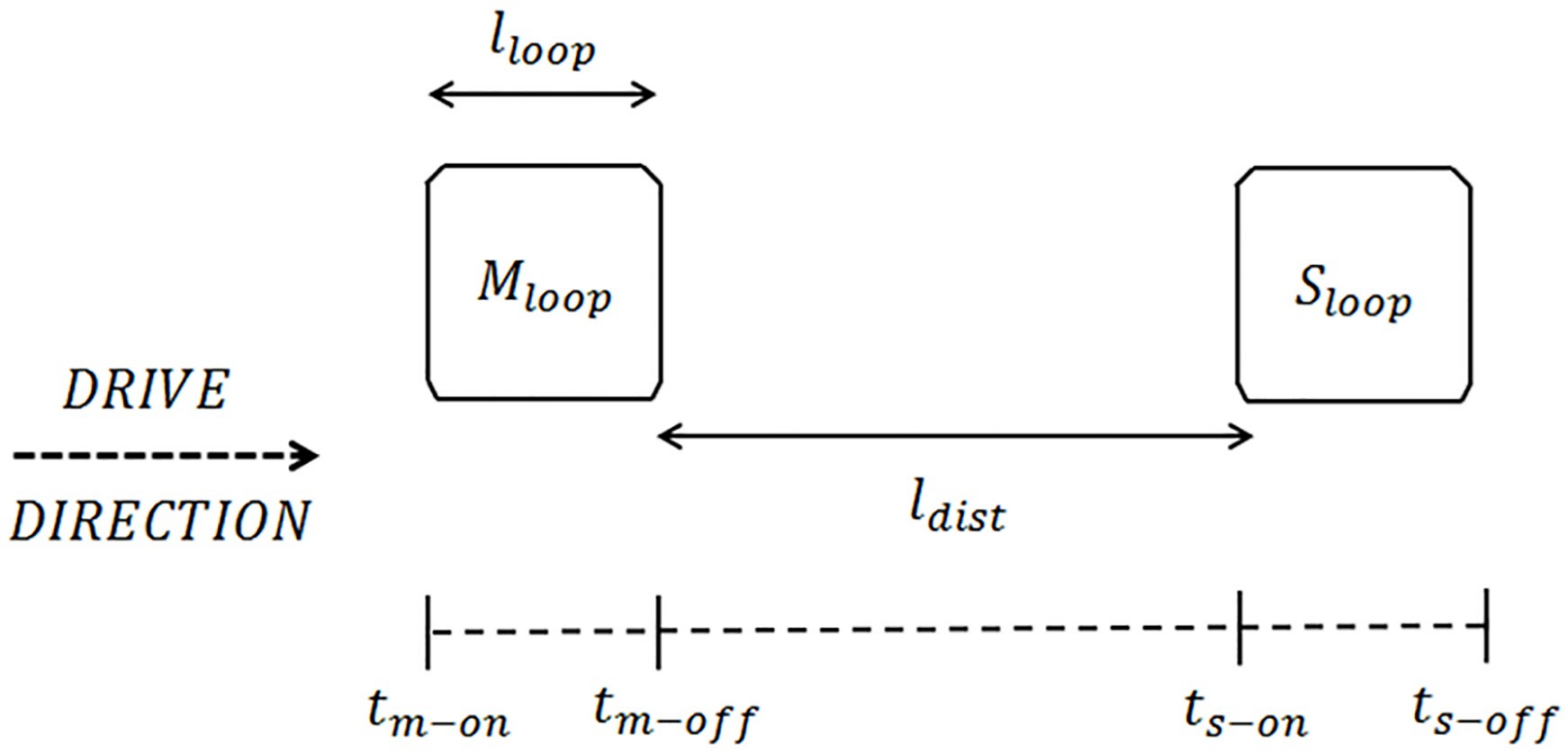
Schematic diagram of dual loop detectors.

Dual-loop detectors, also called speed traps, form a dual-loop system in which two consecutive single magnetic loops, called ‘M loop’ and ‘S loop’ are embedded a small distance apart as shown in Fig 2.

With such a design, when one of them detects a vehicle, timer is automatically started in the dual-loop system and runs until the same vehicle is detected by other loop [19]. In this way, if a vehicle arrives at the first loop (M loop) at *t*_*m*−*on*_ and at the second one (S loop) at *t*_*s*−*on*_, then its speed can be calculated as:

$$Speed=\frac{l_{dist}+l_{loop}}{{(t}_{s-on}-t_{m-on})}$$ (2)

Where,

*l*_*loop*_ = Loop length in meters.

*l*_*dist*_ = Distance between the two loops in meters.

*t*_*m*−*on*_ = Vehicle entry time at first loop in seconds.

*t*_*s*−*on*_ = Vehicle entry time at second loop in seconds.

### Vehicle length

A dual-loop detector is only capable of classifying vehicles according to their lengths. In this manner, the vehicle length can be estimated from its speed and on-times measured by the M and S loops. The on-times for the M and S loops (*On*_*time*−*M*_ and *On*_*time*−*S*_) can be expressed as:

$$\begin{array}{l} On_{time-M}=t_{m-off}-t_{m-on}\\ On_{time-S}=t_{s-off}-t_{s-on} \end{array}$$ (3.1)

Finally, dual-loop algorithm uses Eq 3.2 for vehicle length calculation:

$$L_{vehicle}=[Speed\cdot (\frac{{On}_{time-M}+{On}_{time-S}}{2})]-l_{loop}$$ (3.2)

Since *On*_*time*−*M*_ and *On*_*time*−*S*_ may be different because of possible speed variations over *l*_*loop*_, the mean on-time value is used for calculating vehicle length in order to minimize the estimation error. The loop length term is included in Eq 3.2 because the on-time of a vehicle is measured from the moment the vehicle’s front bumper reaches the leading edge of a single loop to the time its rear end leaves the lagging edge of the loop. Hence the loop length is subtracted from the loop detector’s effective vehicle lengths to give the actual vehicle length. However, it must be taken into account that errors in the measurement of the length would also influence the height of the suspension of the vehicle.

### Direction of traffic

In the same way as the previous parameters, dual-loop detectors can also easily ascertain the direction of circulation. They simply rely on which is the first loop that registers changes in its inductance.

### Vehicle classification

As seen above, loop detectors are based on the impedance variation recorded in the magnetic loops during the passage of vehicles over them, which translates directly into a variation of inductance and frequency. The waveform obtained by plotting the sampled inductance changes is referred to as the magnetic profile or the inductance signature and depends on various vehicle parameters as seen in Fig 1. In this way, many vehicle features can be derived directly or indirectly from its magnetic profile.

**Fig 3:**
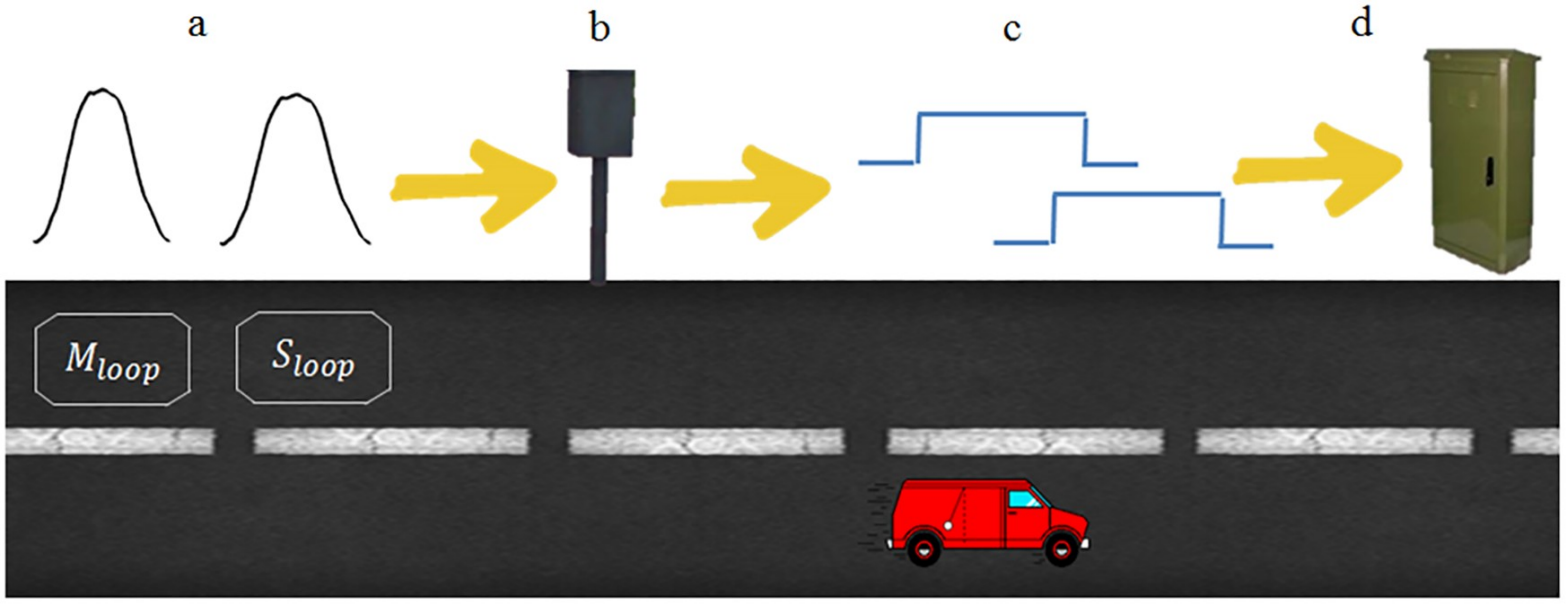
Schematic diagram of how the signals generated by the loops are treated. (a) Real magnetic profiles. (b) Electronics responsible for converting real signals into monostable pulses. (c) Monostable pulses. (d) Urban traffic regulator.

The particularities presented by each type of vehicle have been studied and could be the basis for vehicle classification, but the reality is that this way of classifying vehicles is not used since traffic regulators, responsible for the classification, are not able to process these signals. This is the reason why there are small cabinets located on the sides of the urban roads with the appearance of Fig 3b. They are responsible for transforming signals of the type of Fig 3a into monostable pulses as those shown in Fig 3c, whose duration is the same as the occupation time of the vehicles over the loop. In this way, the traffic regulator can calculate the speed and length of the vehicle and classify it by an estimate of length and not by its magnetic profile.

### Problems associated with the use of single magnetic loops

It has been become clear that for the calculation of all the previous parameters it is required to install two loops, which implies implementing two oscillator circuits, working with two signals, carrying out two civil works and doing two maintenance tasks.

This mainly entails a greater economic investment, which could be reduced practically to 50% with the implementation and start-up of the double loops. In addition, this method of operation in series makes the sensor more vulnerable to failures, since any mismatch in a loop would make it impossible to measure the parameters described above. That is the reason why the vast majority of them are normally only used for counting vehicles, which totally wastes their full potential.

Hence, the objective of this paper focuses on demonstrating the viability and possibilities of this new sensor. For that purpose, it will be detailed how the double magnetic loop improves the performance of the single one by showing how the speed, drive direction and the type of vehicle can be obtained in a simpler and more reliable way.

## Operating principle

**Fig 4:**
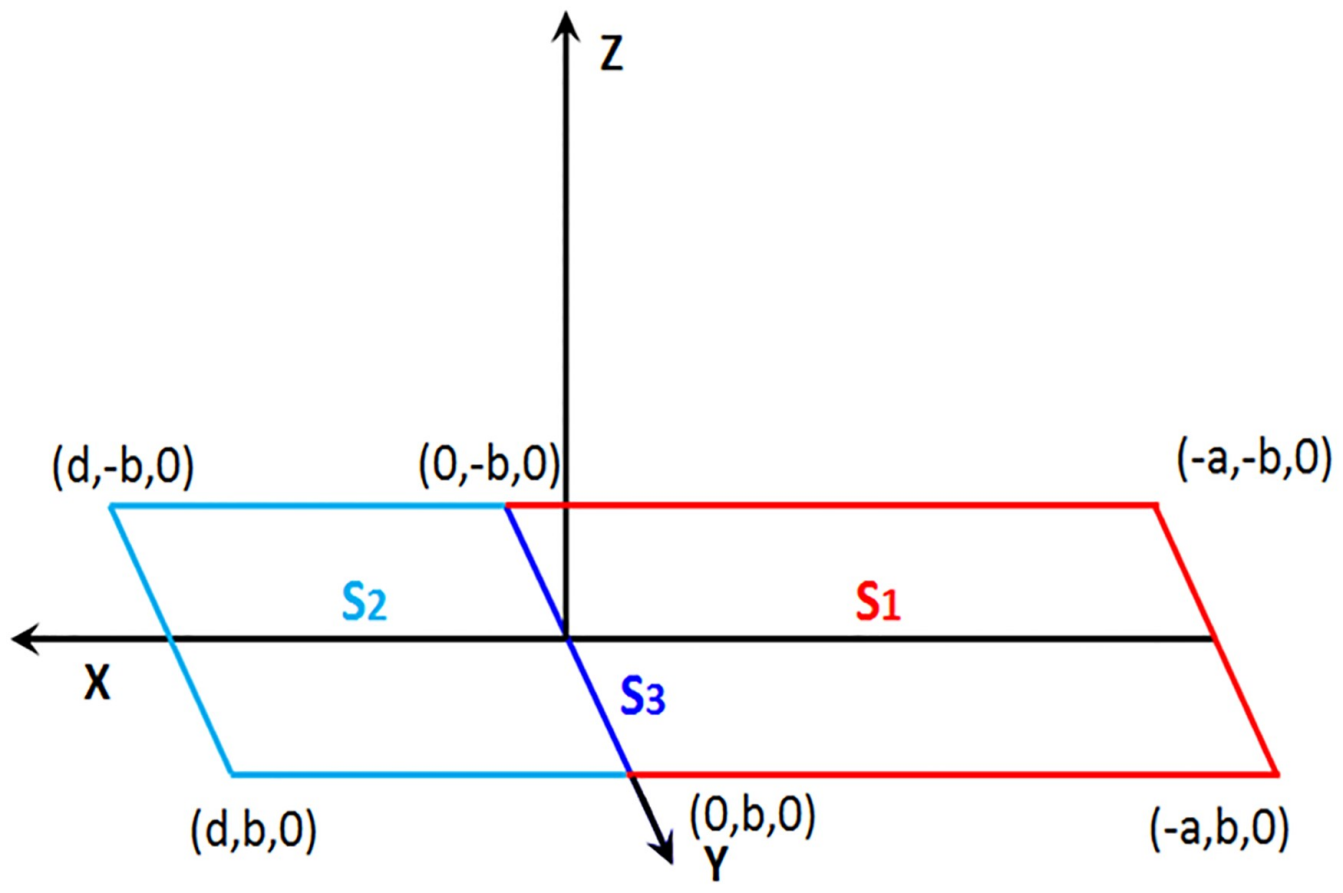
Double magnetic loop presented in three sections.

The design, shape and construction of a rectangular or circular single loop is well known world-wide [31]. Nevertheless, the double loop is still unknown. For this reason we presented in a previous paper a theoretical study to explain the design and peculiarities of the innovative double loops, how to calculate their magnetic field and three different methods to calculate their inductance [29]. In order to have a better understanding of the magnetic profiles generated by these new loops, the space was divided into three sections. The double loop presented had the same appearance as that one of Fig 4.

- The first section, *S*_1_, corresponded to the three red segments located in the plane of the negative values of *X*. Two segments parallel to the *X*-axis with a length of *a* and one segment parallel to the *Y*-axis with a length of 2*b*.
- The second section, *S*_2_, corresponded to the three turquoise segments located in the plane of the positive values of *X*. Two segments parallel to the *X*-axis with a length of *d* and one segment parallel to the *Y*-axis with a length of 2*b*.
- The third section, *S*_3_, corresponded to the blue segment located on the *Y*-axis at *X* = 0, which has a length of 2*b*.

**Fig 5:**
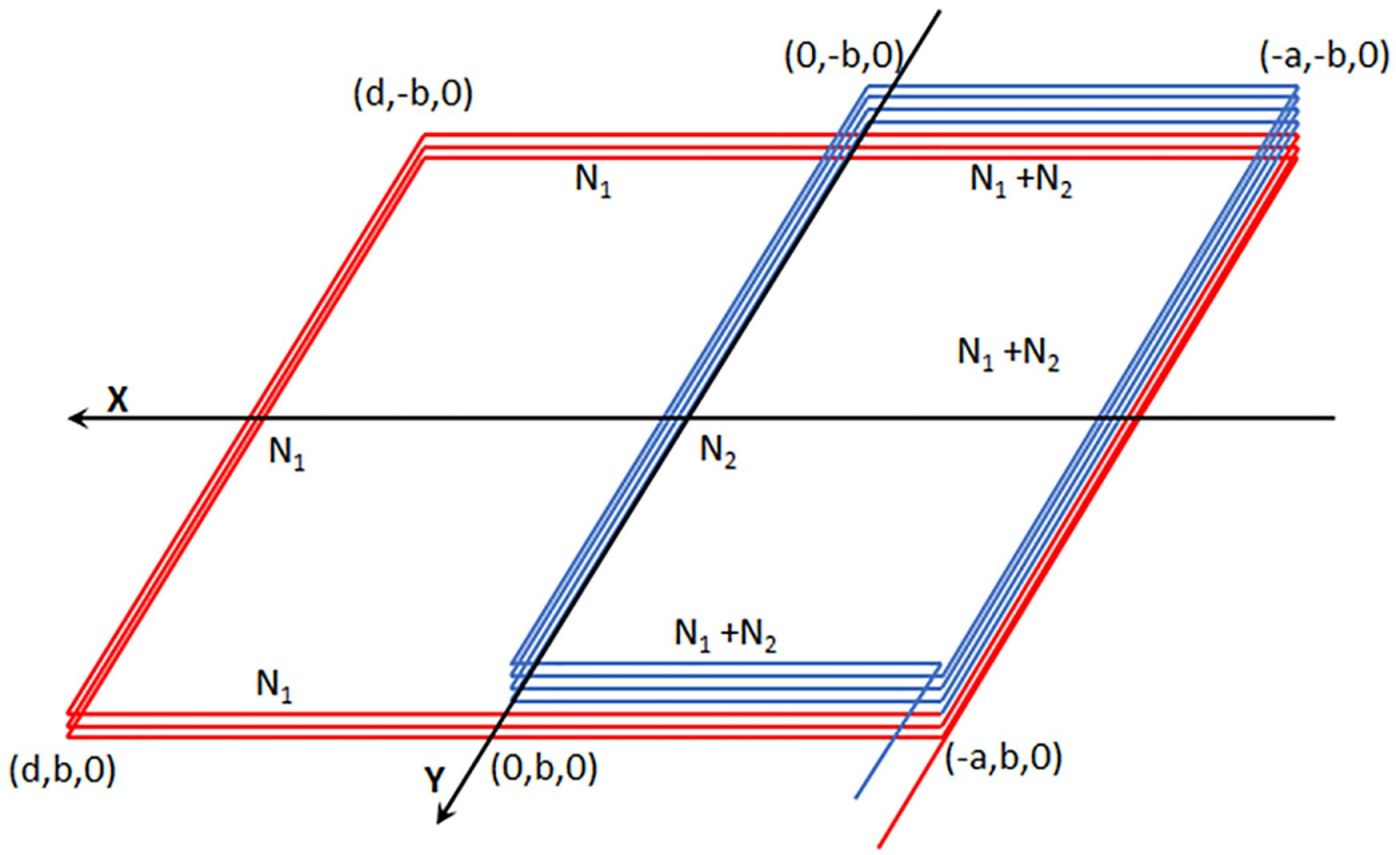
Outline of the double magnetic loop.

Then, the goal was to implement a double loop with an external coil of *N*_1_ turns and, inside and over it, a smaller one of *N*_2_ turns located in the negative half-plane, both with the same direction of circulation. The scheme is shown in Fig 5.

Therefore, in order to discover how the new magnetic profiles will be and check them by means of a simulation program created by ourselves, our work must now focus on calculating the inductance of the loop that will simulates the vehicle and the mutual inductance between this loop and that buried in the pavement.

### Vehicle loop

**Fig 6:**
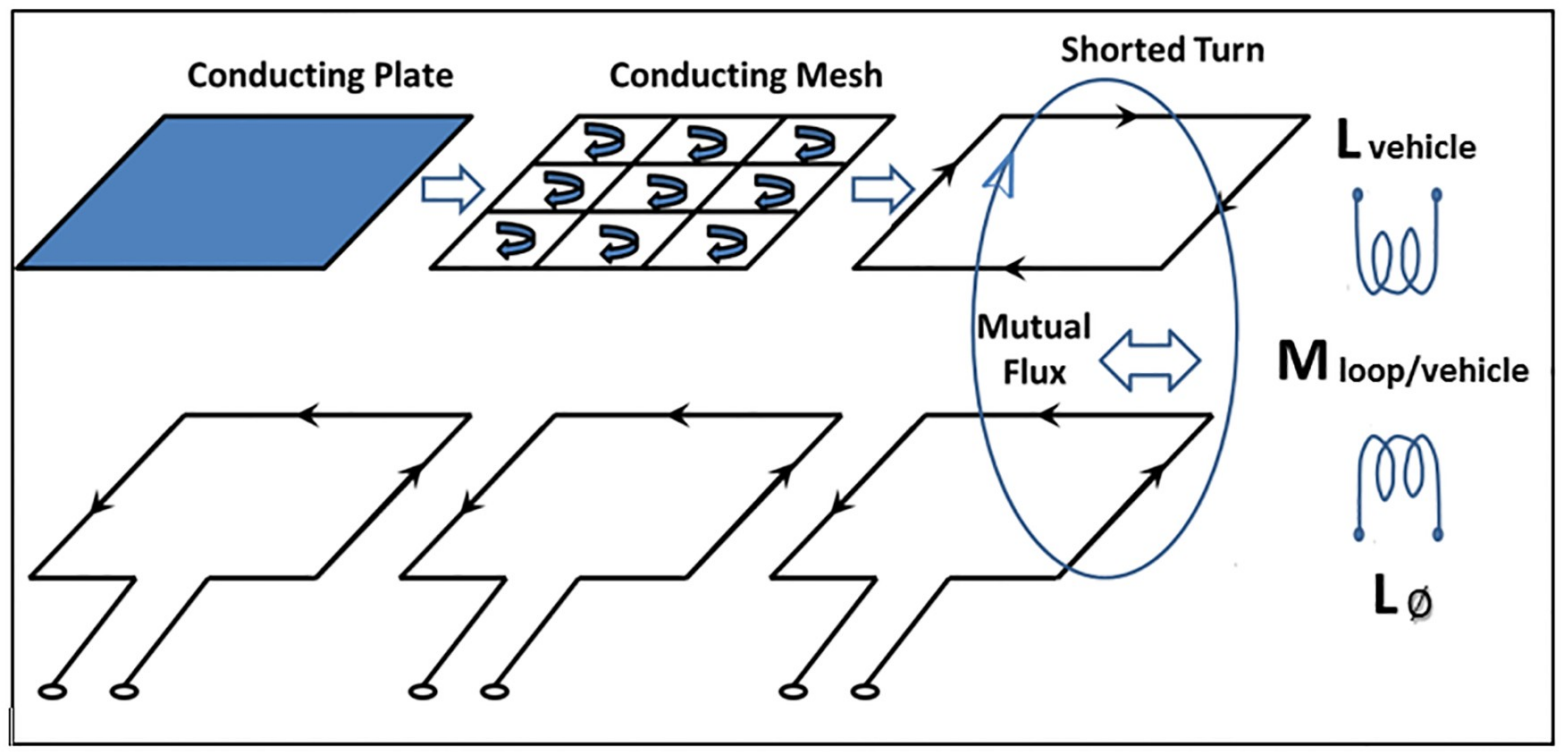
Model of a vehicle passing over the loop.

When trying to simulate the passage of vehicles over loops, vehicles have traditionally been considered as horizontal metal plates. For a long time, different authors [32,33] supported this idea and vehicles were modeled as rectangular metal plates whose width was equal to the width of the vehicle and whose length was equal to the length of the vehicle. Furthermore, these rectangular plates were placed at a certain height from the ground, which corresponded to the average value of the height of the vehicle chassis. The electromagnetic behavior analysis of this modeling could be comparable to the operating mode of the air core transformer shown in Fig 6, where the upper part represents the electric model of the vehicle passing over the magnetic loop and the lower part represents the loop buried in the pavement.

In this way, we could use any of the existing expressions in the specialized literature for rectangular loops [34–36], but among them, one of the most common and reliable responds to:

$$L\approx \frac{\mu_{0}\mu_{r}}{\pi }[-4(a+b)+4\sqrt{a^{2}+b^{2}}-2b\;\text{ln}(\frac{b+\sqrt{a^{2}+b^{2}}}{a})-2a\;\text{ln}\left(\frac{a+\sqrt{a^{2}+b^{2}}}{b}\right)+2b\;\text{ln}\left(\frac{4b}{r}\right)+2a\;\text{ln}\left(\frac{4a}{r}\right)]$$ (4)

Where:

- *μ*_0_ = Vacuum permeability.
- *μ*_*r*_ = Relative permeability of the medium.
- *r* = Conductor radius.
- 2*a* = Length of the equivalent loop to the vehicle.
- 2*b* = Width of the equivalent loop to the vehicle.

### Mutual inductance

Fig 6 showed the magnetic coupling between a loop and a conductor that forms a closed loop, which functions as a transformer with an air core. Then, the mutual inductance between both elements could be defined as the current flowing in one coil and induces a voltage in the adjacent ones.

The amount of mutual inductance that links one coil to another depends very much on the relative positioning of the two coils. If one coil is positioned next to the other one so that their physical distance apart is small, then nearly all of the magnetic flux generated by the first one will interact with the coil turns of the second one, inducing a relatively large electromagnetic field and therefore producing a large mutual inductance value.

Therefore, since our system will have a fixed loop and another one that will move over the previous one, the mutual inductance between the primary circuit, the double loop, and the secondary circuit, the conductor, will be given by:

$$M_{loop/vehicle}=\frac{N_{S}\cdot \emptyset }{I}$$ (5)

Being:

- *M*_*loop*/*vehicle*_ = Mutual inductance between the primary and the secondary circuit in Henry.
- *N*_*S*_ = Number of turns of the secondary one. (For a closed-loop conductor its value is 1).
- ∅ = Magnetic flux in Webers generated by the magnetic field created by the loop that is perpendicular to the area formed by the closed-loop that represents the vehicle.
- *I* = Current intensity that circulates through the loop in Amperes.

**Fig 7:**
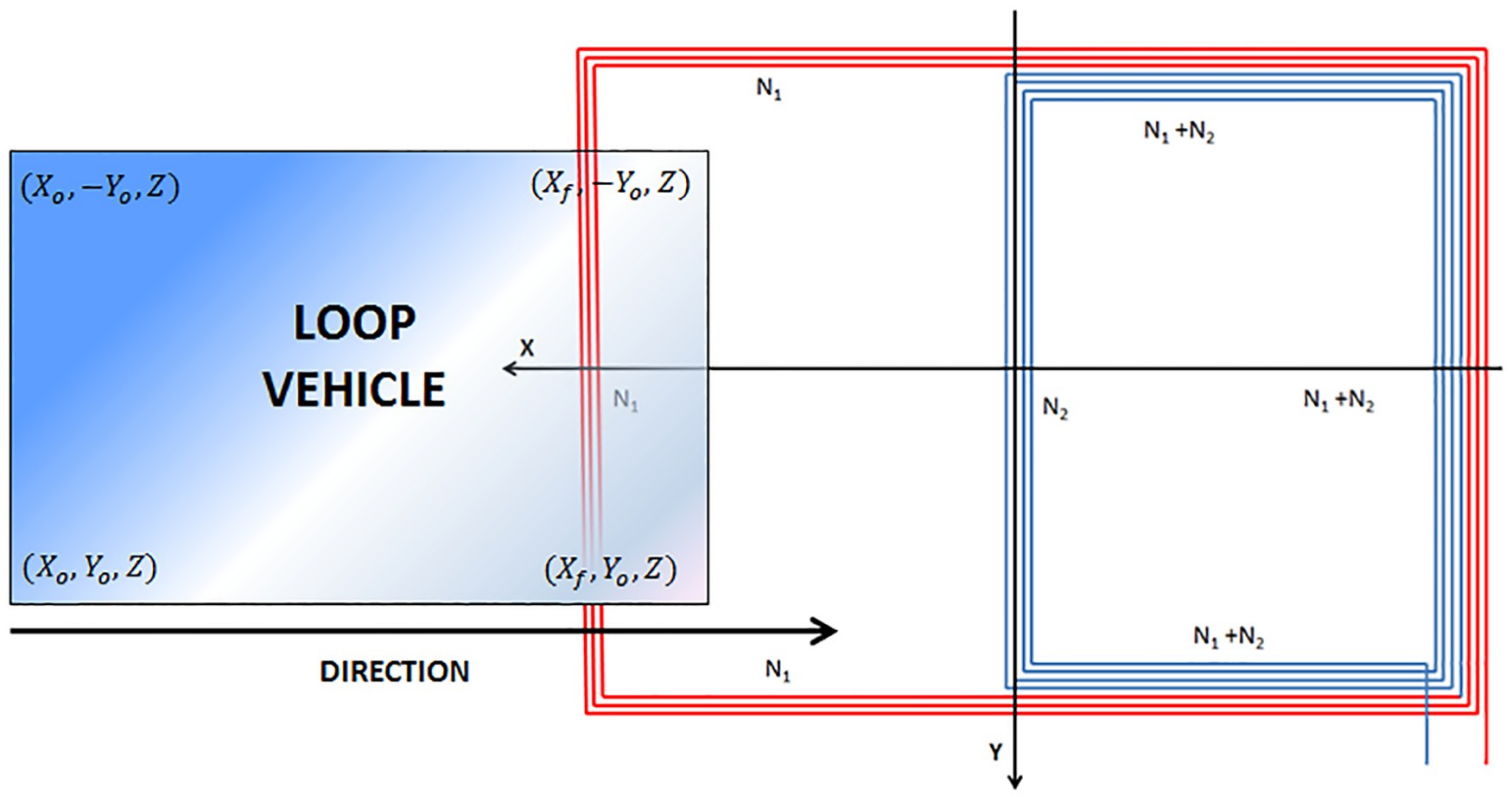
Equivalent vehicle model passing over the loop at a specific moment of time.

For the calculation of this mutual inductance, it will be necessary to calculate the magnetic field generated by the loop buried on the road at each point of the surface of the equivalent vehicle loop. For this purpose, several authors, including us, have presented different methods for calculating the magnetic field generated by magnetic loops, for both single [36], and double loops [29]. Nevertheless, if it is considered that the chassis of the vehicle is parallel to the roadway at a certain height, only the component of the magnetic field along the *Z*-axis should be taken into consideration for the calculation of the flux [29]. Fig 7 shows a schematic diagram of our proposal.

When considering the whole set as shown in Fig 7, a mathematical model that represents this behaviour is required. We have chosen one of those shown in the Annex E of Traffic Detector Handbook [31], and according to this model, the equivalent impedance of the previous circuit when a vehicle passes over a loop would be given by Eq 6.

$$Z_{1}=\frac{Z_{11}Z_{22}-Z_{21}^{2}}{Z_{22}}$$ (6)

Where:

- Z_1_ = Equivalent impedance of the whole set.
- Z_11_ = Impedance of the loop buried on the road, assuming it is isolated.
- Z_22_ = Impedance of the equivalent vehicle loop, assuming it is isolated.
- Z_21_ = Mutual impedance between both.

It should be noted that for the model considered, all the impedances will be inductances, regardless of whether they are self-inductances or mutual inductances. Consequently, Z_1_ will also be an inductance measured in Henry.

*Z*_11_ represents the impedance of the loop buried on the road when it is considered isolated and there are no vehicles in the vicinity. It is a constant value regardless of whether or not there is a vehicle over the loop. It will be represented as *L*_∅_.

*Z*_22_ is the impedance of the loop that simulates the vehicle, whose expression will be similar to that shown in Eq 4. In this case, 2*a* and 2*b* will be respectively the length and width of the vehicle and *r* will be the thickness of the metal plate with which has built the chassis of the vehicle (it is assumed a value of 1 *mm*). Its value will also remain constant for a given vehicle model and therefore will be a parameter that will help in its classification. It will be represented as *L*_*vehicle*_.

*Z*_21_ represents the mutual inductance between the loop buried on the road and the loop that models the vehicle. It was represented as *M*_*loop*/*vehicle*_ in Eq 5. This term will be the only one that depends on the type and position of the vehicle and, therefore, it will provide information on the type, speed and direction of the vehicle.

Consequently, reformulating Eq 6, the equivalent impedance will be an inductance *L*_*eq*_ whose value will be given by the expression:

$$L_{eq}=\frac{L_{\Phi }L_{vehicle}-M_{loop/vehicle}^{2}}{L_{vehicle}}$$ (7)

Thus, the oscillation frequency of the circuit will be given by the aforementioned Eq 1.

In Eq 7 all terms remain constant regardless of the position of the vehicle except *M*_*loop*/*vehicle*_. Then, if we call *f*_0_ at the oscillation frequency of the circuit when there are no vehicles over the loop (*M*_*loop*/*vehicle*_ = 0):

$$\frac{1}{f}=\frac{1}{f_{0}}-\frac{M_{loop/vehicle}^{2}}{kL_{vehicle}}$$ (8)

From Eq 8 it can be assumed that:

$$\frac{M_{loop/vehicle}^{2}}{kL_{vehicle}}=\frac{f-f_{0}}{ff_{0}}\approx \frac{\Delta f}{f_{0}^{2}}$$ (9)

This approximation is acceptable since the frequency of oscillation of the systems based on loops takes values around 10^5^ *Hz* and the deviation in frequency does not usually exceed 500 *Hz*. Accordingly, we could approximate with these values the deviation in frequency suffered by the oscillator circuit by the following expression:

$$\Delta f=f_{0}^{2}\frac{M_{loop/vehicle}^{2}}{kL_{vehicle}}$$ (10)

To operate with exact values instead of working with the oscillation frequency, we can also work with the signal periods (*T*). In this case, the last expression would be converted to:

$$T=T_{0}-\frac{M_{loop/vehicle}^{2}}{kL_{vehicle}}$$ (11)

Then:

$$\Delta T=-\frac{M_{loop/vehicle}^{2}}{kL_{vehicle}}$$ (12)

In both the approximate and exact expressions it is clear that the magnetic profile of any vehicle which passes over the loop will be proportional to the square of the mutual induction and inversely proportional to the self-induction of the vehicle. This self-induction will be a constant parameter for each vehicle while the mutual induction will be a parameter that will depend on the relative position between this and the loop.

Moreover, from this expression it becomes clear that it is impossible to determine the type of vehicle and its speed in an exact way with a single loop, since there is two unknown data (*M*_*loop*/*vehicle*_ and *L*_*vehicle*_) with the variation of a single parameter (Δ*f*), which is obtained from the magnetic profile left by the vehicle. Hence, there are generally two loops per lane and the way to classify vehicles and calculate their speed by using single loops is done by means of approximations. In this manner, in order to see what the advantages offered by the double turns are, in the following point the inductance signatures generated by the passage of vehicles over them, both at the theoretical and at the experimental level, will be analysed.

## Magnetic profiles generated by the passage of vehicles over double loops

With the criterion and nomenclature used in Figs 5 and 7, the flux ∅ shown in Eq 5 could be obtained by a numerical integration along the surface of the vehicle chassis. The accuracy of this calculation will depend on the number of points considered for the integration. Then, if we consider a number of points *N*_*px*_ according to the *X*-axis and a number of points *N*_*py*_ according to the *Y*-axis, the surface differentials will be established as *dS* = *dx*_*v*_*dy*_*v*_, where:

$$dx_{v}=\frac{X_{0}-X_{f}}{N_{px}}\;dy_{v}=\frac{2Y_{0}}{N_{py}}$$ (13)

Under these conditions, the expression of the flux through the equivalent loop of the vehicle at each instant would be:

$$\emptyset_{\;}=\sum_{n=1}^{N_{px}}\sum_{m=1}^{N_{py}}B_{K}(X_{0}+ndx_{v},Y_{0}+mdy_{v},z)dy_{v}dx_{v}$$ (14)

Where *B*_*K*_ is the component of the magnetic field generated by the double loop buried in the pavement perpendicular to the plane of the loop that represents the vehicle, whose expression can be found in [30].

It must be noted that with the aim of simplifying, it has been considered that the vehicle moves centered with respect to the Y-axis. If not, the calculation would only be modified by the points where the summation is applied. However, this is mostly true in real environments.

To implement this theoretical model, an application has been developed both in VisualBasic and Matlab. This performs all the processes of calculation, graphical presentation of results and storage of them in a file. In this way, a series of parameters must be introduced before simulating. These are:

- The geometrical characteristics of the loop: dimensions according to the *X* and *Y*-axes.
- The type of copper conductor used, its radius and the current that will flow through it.
- The spacing between turns [29].
- The number of points used to calculate the self-induction of the loop according to the *X* and *Y*-axes for the numerical integration (although if these ones are not introduced, the system assigns the values that proved to be optimal [29,36] by default).
- The number of turns of the loop. For single loops *N*_2_ = 0.

In relation to the vehicles, this software allows to model them as rectangular metal plates with the dimensions of their chassis according to the model explained in Fig 7. Therefore, it will also be necessary to indicate the characteristics of the vehicle referring to its dimensions according to the three axes (length, width and height of the chassis over the asphalt), which is usually taken as the arithmetic mean of the height vehicle. Furthermore, the trajectory traversed by the center of the vehicle in the three directions of the space (*X*_*o*_, *X*_*f*_, *Y*_*o*_, *Z*) and the speed at which it does must also be entered.

After introducing all the above data, the last values required for the simulation are the electrical characteristics of the components that constitute the oscillating circuit where the loop is incorporated. The oscillator circuit model is shown in [30], where *V*_*CC*_, *V*_*TH*_, *V*_*TL*_ and *R* determine the value of *k* in Eq 1.

**Fig 8:**
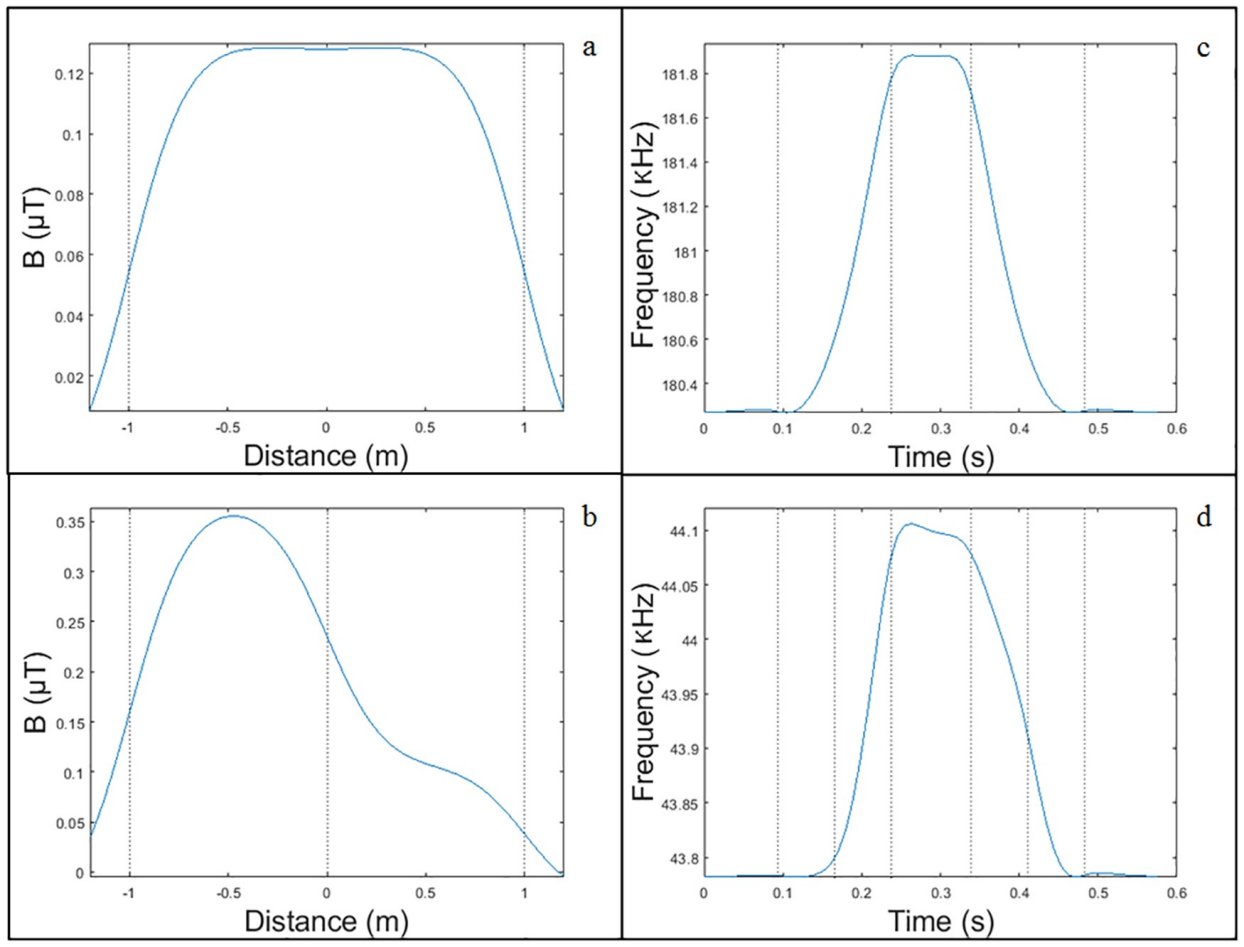
Single loop versus double loop. (a) Magnetic field produced by a simple loop. (b) Magnetic field produced by a double loop. (c) Magnetic profile produced by a simple loop. (d) Magnetic profile produced by a double loop.

Thus, in order to analyze the advantages offered by the double loops compared to the single ones, first a simulation will be carried out for both detectors in which the magnetic field that these produce and the magnetic profiles generated after the passage of a vehicle will be shown. The vehicle chosen was a Citroën-AX of 3.4 *m* long, 1.5 *m* wide and with a mean height of the chassis of 0.5 *m* above the ground, which passed at a speed of 50 *Km*/*h* over them. In both cases, the loop had an external dimension of 2 *x* 2 *m*. Fig 8 shows the result after entering the parameters of Tables 1, 2 and 3 into the software designed.

**Table 1:** Loop characteristics entered in simulation.

Loop Characteristics	Single	Double
N_EGATIVE X-SIDE (METERS)_	1	1
P_OSITIVE X-SIDE (METERS)_	1	1
Y_AXIS (METERS)_	1	1
C_ABLE RADIO (MILLIMETERS)_	0.75	0.75
S_EPARATION BETWEEN TURNS (MILLIMETERS)_	1.9	1.9
I_NTENSITY (AMPS)_	0.1	0.1
N_UMBER OF TURNS_ (*N*_1_)	3	3
N_UMBER OF TURNS_ (*N*_2_)	0	5
*μ*_*r*_	1	1

**Table 2:** Vehicle characteristics entered in the simulation.

Oscillator Characteristics	Single/Double
X_0 (METERS)_	4
Y_0 (METERS)_	0
Z_0 (METERS)_	0.5
X_F (METERS)_	-4
Y_F (METERS)_	0
Z_F (METERS)_	0.5
V_EHICLE SPEED (KILOMETERS/HOUR)_	50
C_ALCULATION POINTS_	50

**Table 3:** Oscillator characteristics entered in the simulation.

Oscillator Characteristics	Single/Double
V_CC_ (V)	4.4
V_TH_ (V)	1.8
V_TL_ (V)	0.95
R (Ω)	15

From Fig 8 it can be concluded that:

- The magnetic field produced by a simple loop is symmetrical.
- The magnetic field produced by a double loop is not symmetrical since it increases significantly due to the smaller inner loop located on the right extreme.
- The magnetic profile generated by the simple loop is also symmetric.
- The magnetic profile generated by the double loop is not symmetrical and has many more slope changes than the previous one.
- Passenger cars usually have the motor on the front part, which makes the metal area in this part bigger than in the rest of the vehicle. This is equivalent to a lower height in the front, which implies a greater mutual inductance, and that is the reason why the highest peak of the magnetic profile is at the beginning of both profiles.

**Fig 9:**
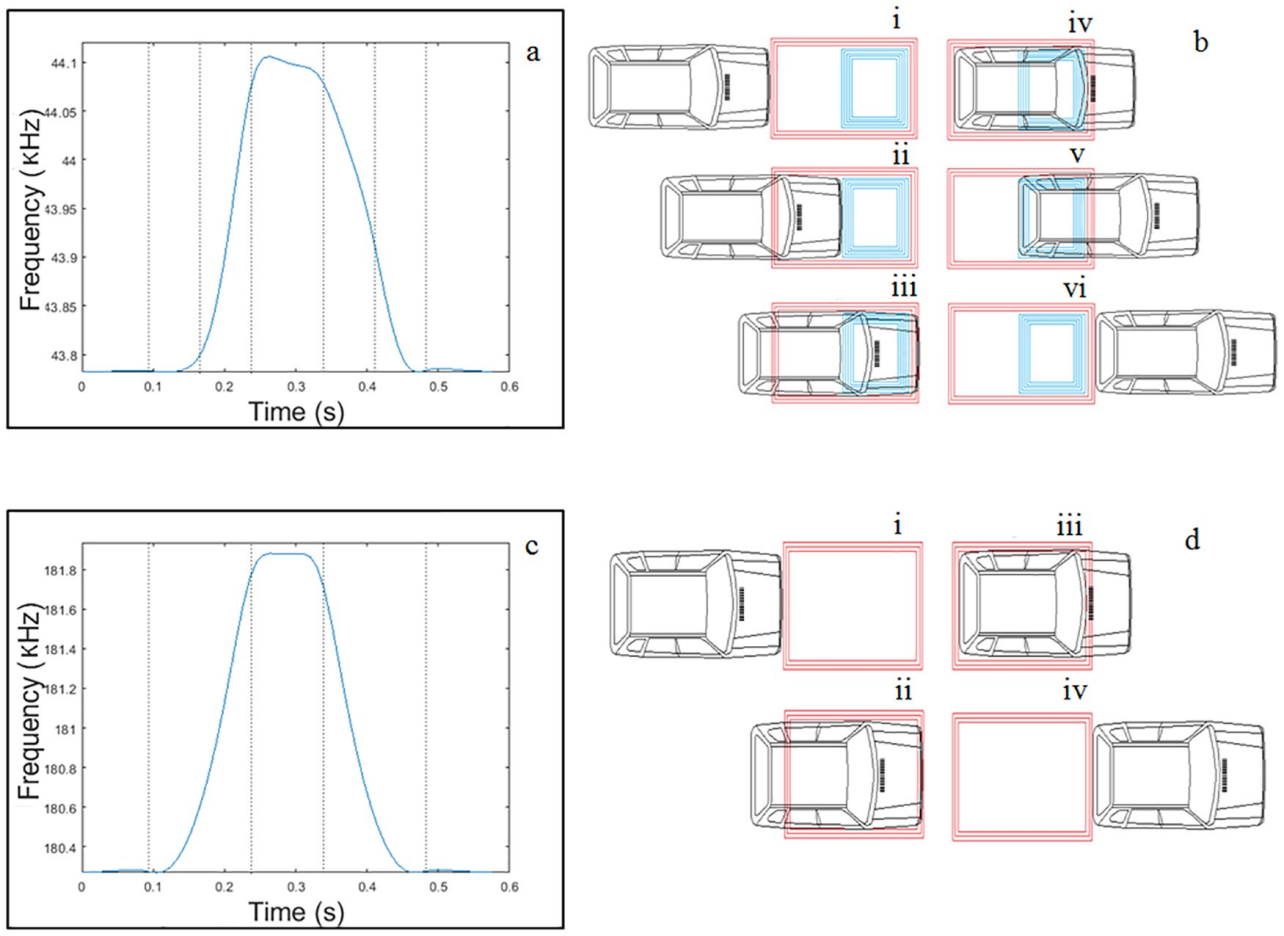
Relationship between the changes of the magnetic profile and the position of the vehicle with respect to the loop. (a) Double loop oscillation frequency. (b) Different vehicle positions over the double loop. (c) Single loop oscillation frequency. (d) Different vehicle positions over the single loop.

Moreover, it must be pointed out that we can observe that several broken lines have been added to Fig 8. These lines try to improve the interpretation and understanding of existing information. In this way, Fig 9 explains the relationship between the different positions of the vehicle over the loop and the magnetic profiles generated.

As can be seen in Fig 9, when a magnetic profile is generated after the passage of a vehicle over a double loop, six reference points are obtained:

- The first one is when the vehicle comes into contact with the large loop of *N*_1_ turns (*S*_2_).
- The second one is when the vehicle begins to enter the small loop of *N*_1_ + *N*_2_ turns (*S*_3_).
- The third one is the instant in which the vehicle has begun to completely occupy both loops.
- The fourth one is when the vehicle starts to leave the first loop of *N*_1_ turns (*S*_2_).
- The fifth one is when the vehicle starts to leave the small loop of *N*_1_ + *N*_2_ turns (*S*_3_).
- The sixth is when the vehicle has completely left the double loop.

Nevertheless, when a magnetic profile is generated after the passage of a vehicle over a single loop, only four reference points are obtained. This can be seen in Fig 9c and 9d.

- The first one is when the vehicle comes into contact with the loop.
- The second one is the instant in which the vehicle has begun to occupy the whole loop.
- The third one is when the vehicle starts to leave the loop.
- The fourth is when the vehicle has completely left the loop.

After observing both graphs, the first thing that should be highlighted is that the magnetic profile generated by the single loop is symmetric and therefore provides much less information than that one generated by the double loop. Thus, with such a magnetic profile, it is impossible to determine the direction of circulation. Moreover, since there are only four reference points, the vehicle speed can not be obtained exactly. This is the reason why, generally, either estimates are used or two loops per lane are placed. Thus, in the following sections it will be shown all the advantages offered by this type of loops by showing how the double loop is able to obtain all these parameters from these magnetic profiles with very small error margins.

## Data collection

To verify the goodness of the model, a double loop with the same previous values was implemented and installed by our research group in the facilities of the Polytechnic University of Valencia. Moreover, our research team (Group of Traffic Control Systems) had a Citroën-AX vehicle with the same characteristics indicated in the theoretical model, which was driven over the loop many times. The magnetic profile was recorded as the deviation of the oscillation frequency from the value in the absence of vehicle by SCT-CEM-4 device. This equipment is an improved version of the SCT-IL v2.0 system developed by the Traffic Control Systems Group of the ITACA Institute of the Polytechnic University of Valencia, which is patented with the application number P200401111 and whose details are given in [30].

In order to maximize the understanding and to ensure its reliability, the signal was normalized between a minimum value equal to zero and a maximum value equal to 100. This is:

$$f_{n}=\frac{f(t)-f_{o}}{{\left(f(t)-f_{o}\right)}_{max}}$$ (15)

where *f*(*t*) is the frequency at the time t, *f*_*o*_ is the oscillation frequency value when there is no vehicle over the loop and (*f*(*t*) − *f*_*o*_)_*max*_ is the oscillation frequency maximum deviation observed when the vehicle passes over the loop.

These tests were performed at 666 samples per second, although the sampling frequency is selectable between 250, 400, 500 or 666 samples per second. The type of oscillator used for the connection of the loops is based on the NAND gates with Schmitt-trigger [30].

Moreover, it works with 2 DSPs capable of making a classification of vehicles in real time.

**Fig 10:**
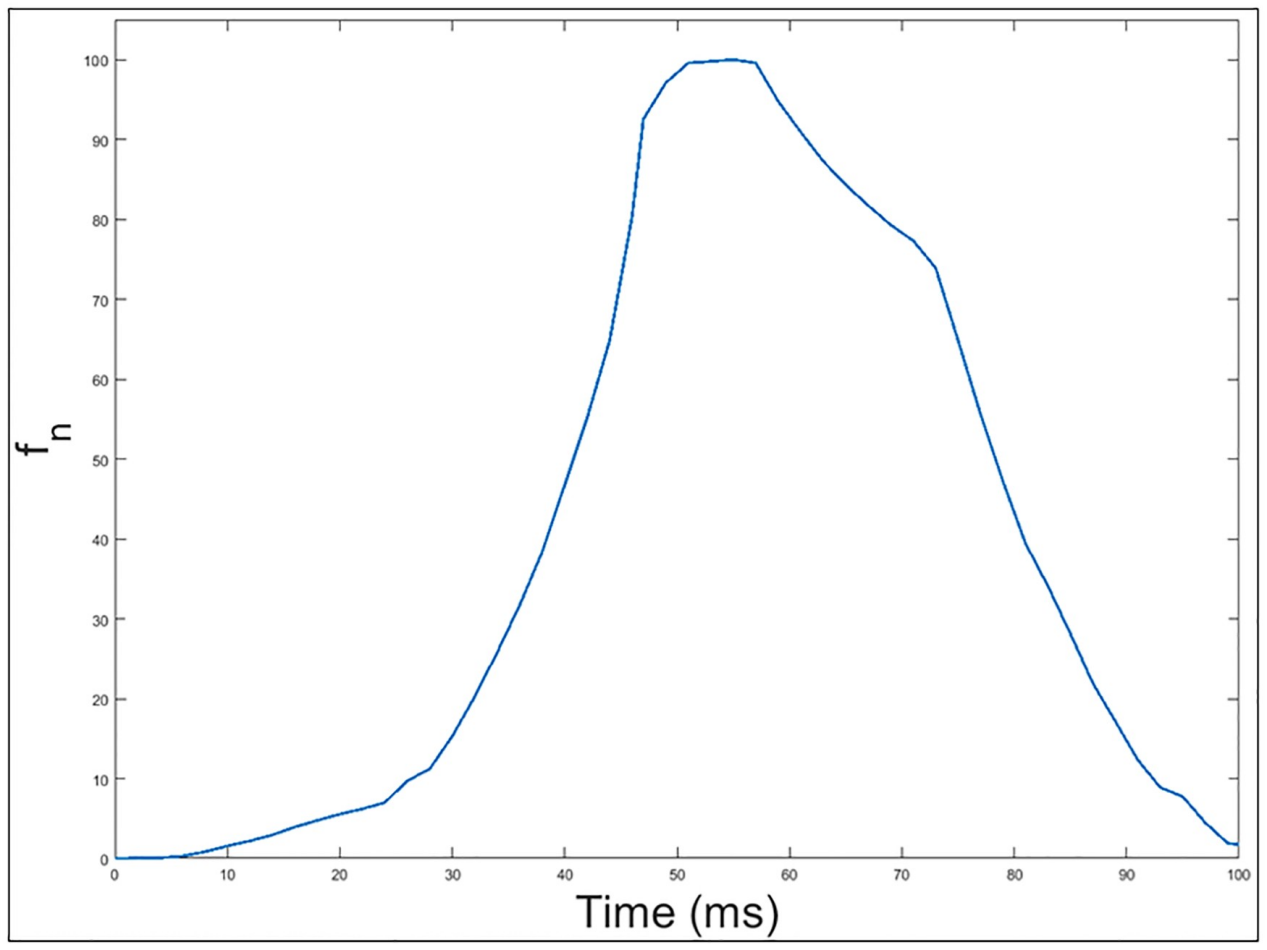
Magnetic profile generated by the passage of a Citroën-AX vehicle over a double loop.

With this configuration, the recorded magnetic profile of the Citroën-AX vehicle is shown in Fig 10.

The resemblance between the theoretical and experimental graphs is remarkable. However, the presence of slope changes each time the vehicle reaches each of the sections of the double loop is the most relevant information of these new magnetic profiles. In fact, these slope changes are those that are intended to be used to determine the vehicle’s speed and length and direction, which will be shown in the following sections.

## Vehicle speed and vehicle length from magnetic profiles

**Fig 11:**
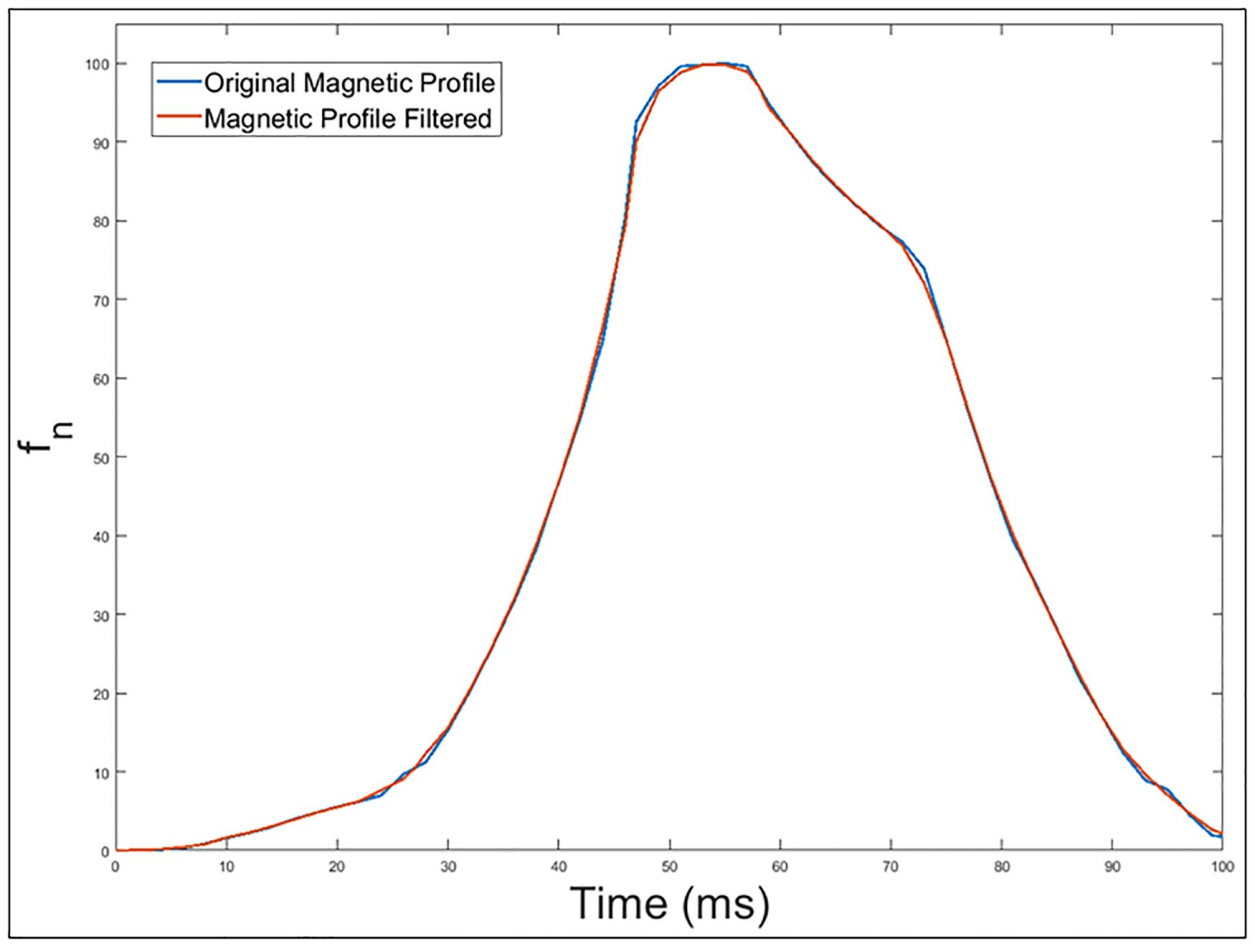
Original and filtered magnetic profile generated by the passage of a Citroën-AX vehicle over a double loop.

For the determination of the vehicle speed and its length from its magnetic profile, the following methodology is proposed:

- Performing a filtering of the signal in order to avoid irregularities as shown in Fig 11.
- Obtaining the normalized square root of the filtered magnetic profile generated by the passage of the vehicle over the double loop.

**Fig 12:**
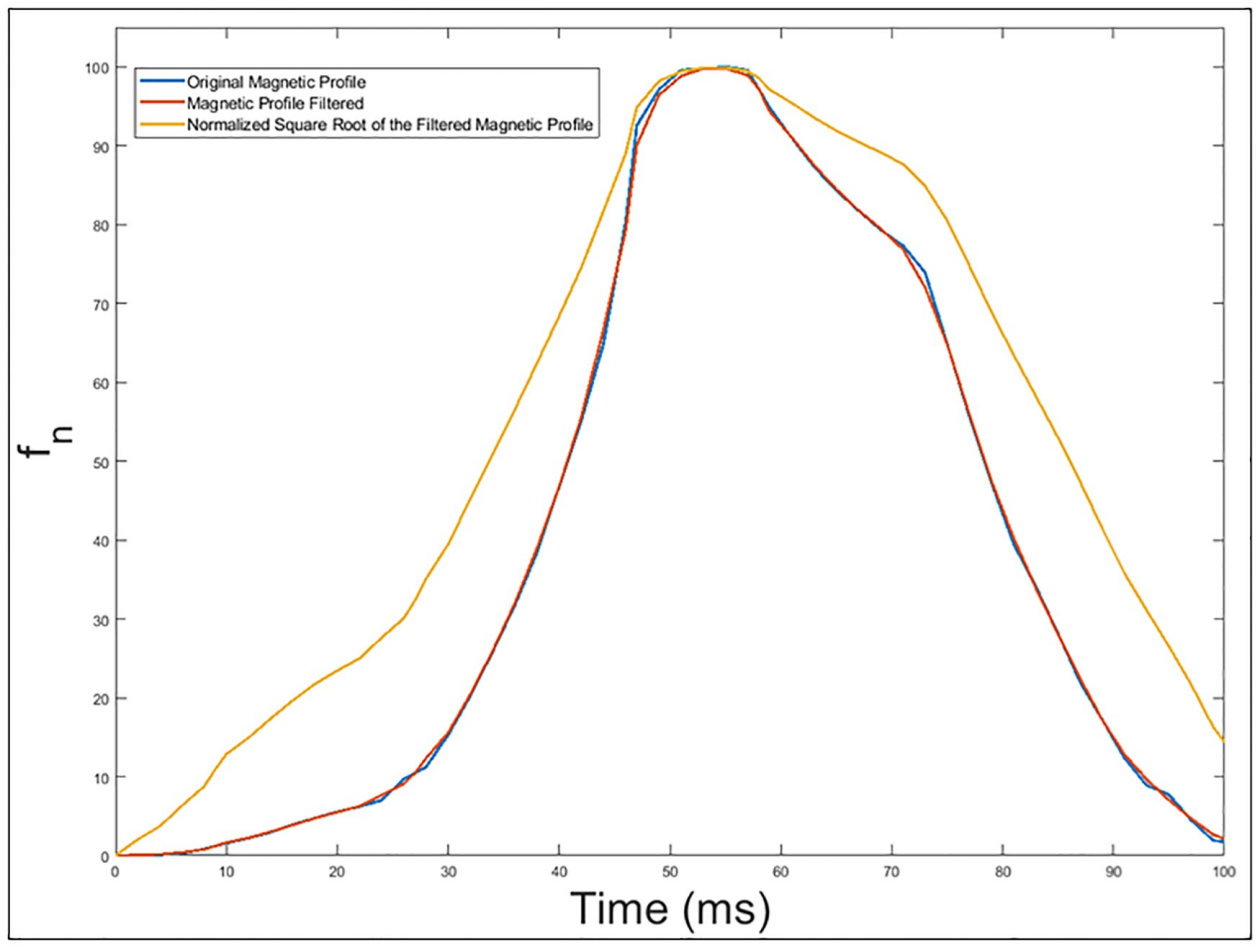
Original and filtered magnetic profile generated by the passage of a Citroën-AX vehicle over a double loop and the normalized square root of the filtered frequency deviation.

The square root of the frequency deviation have been introduced in Fig 12 because as seen in Eq 10, the frequency deviation is proportional to the square of the mutual inductance, which depends directly on the relative position between the vehicle and the loop buried in the pavement.

**Fig 13:**
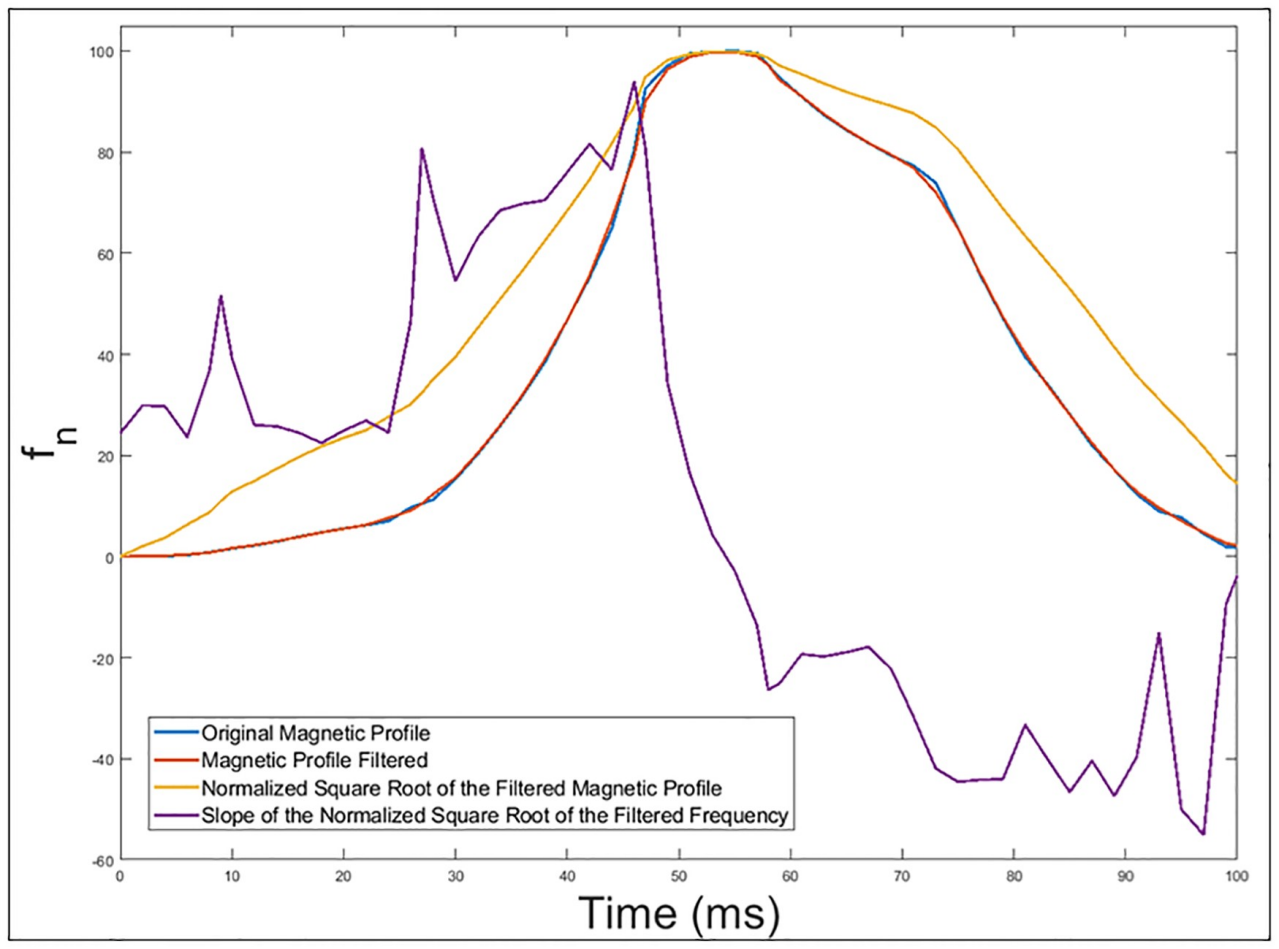
Calculation of the slope of the normalized square root of the filtered frequency deviation.

**Fig 14:**
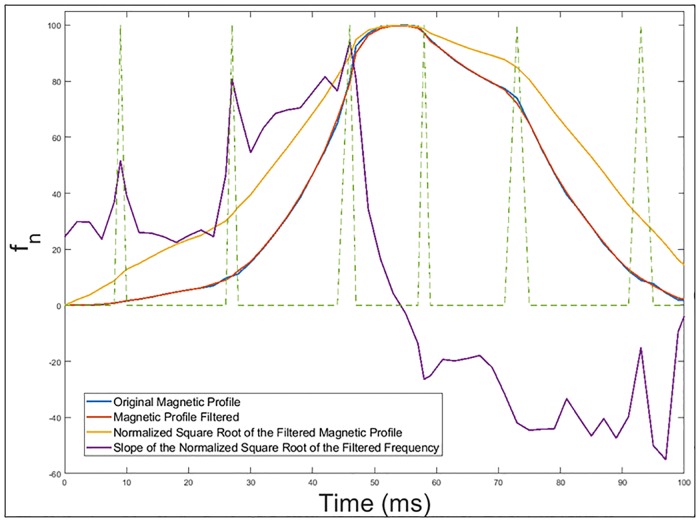
Points where there is a sharp change in the slope of the normalized square root of the filtered frequency deviation.

- Calculation of the slope of the normalized square root of the filtered frequency deviation. These slope changes will be obtained as the numerical derivative of the inductance signature. These slopes superposed to the previous figure can be seen in Fig 13. This procedure is also used for axle detections in current studies related with inductive loops [26].
- Determination of the points where there is a sudden change in the slope of the normalized square root. Fig 14 shows these points of abrupt slope change.

These points are obtained by detecting when the absolute value of the derivative of the square root exceeds a certain threshold. The vast majority of these slope changes should represent the arrival or departure of the vehicle to each of the three sections of the double loop mentioned above and showed in Fig 9b, although some intermediate points can also be obtained.

### Vehicle speed

In this way, as the separation between the different sections of the loop is known by design, the vehicle speed can be obtained as the quotient between the distance of the section and the time elapsed between the slope changes. In the case of Fig 14, the different sections were separated by 1 *m* and there were six points of abrupt slope change. Table 4 shows the relationship between abrupt changes in slope and time.

- P_1_ indicates the moment of time when the front end of the vehicle reaches the positive section of the loop (*S*_2_).
- P_2_ represents the instant when the front part of the vehicle reaches the intermediate section of the double loop (*S*_3_).
- P_3_ corresponds to the first instant in which the loop is fully occupied (*S*_1_ has been occupied).
- P_4_ would intuitively indicate the instant in which the rear part of the vehicle begins to leave *S*_2_, but this is not the case. It is an intermediate point while the loop is fully occupied. This can be corroborated by seeing Fig 9a.
- P_5_ actually represents the instant in which the rear part of the vehicle begins to leave the positive section of the loop (*S*_2_).
- P_6_ marks the instant of time when the rear part of the vehicle passes over the section of the loop located on the Y axis (*S*_3_).

**Table 4:** Points of abrupt slope change.

Points	Time (ms)
P_1_	9
P_2_	27
P_3_	46
P_4_	58
P_5_	73
P_6_	93

In this way, the speed can be obtained by using different points.

With P_1_ and P_2_: *Speed*_*vehicle*1_ = 1000 / (27 − 9) = 55.5 *km* / *h*
With P_2_ and P_3_: *Speed*_*vehicle*2_ = 1000 / (46 − 27) = 52.6 *km* / *h*
With P_5_ and P_6_: *Speed*_*vehicle*3_ = 1000 / (93 − 73) = 50 *km* / *h*

By averaging these three values, the final vehicle speed obtained would be:

$$V_{vehicle}=52.7\;km\;/\;h.$$

As can be seen, this average speed is practically the same used for the simulation. Nevertheless, it must be noted that the most reliable points are those that have been chosen in the previous case, since displacements of the peaks may appear in the central zone due to the geometry of the vehicles.

### Vehicle length

To obtain the size of the vehicle, it is necessary to see how long it takes to pass over each of the representative points of the different sections of the double loop. For example, as seen in Fig 9b, the time that the vehicle takes to pass over the positive section of the loop will be the difference between P_5_ and P_1_, but the time to pass over the negative section of the loop will be the difference between P_6_ and P_2_. In this way, considering the calculated speed, the length of the vehicle will be obtained as the product of this average by the time difference in which the vehicle is over certain sections of the loop.

For points P_5_ and P_1_:

$$L_{vehicle1}=V_{vehicle}\;(t_{5}\;-\;t_{1})=52.7\;\cdot \;(73-9)\;/\;1000=3,4\;m$$

For points P_6_ and P_2_:

$$L_{vehicle2}=V_{vehicle}\;(t_{6}\;-\;t_{2})=52.7\;\cdot \;(93\;-\;27)\;/\;1000=3,4\;m$$

Therefore, averaging both values in the same way as with the speed, the vehicle length could be finally obtained. However, as both values coincide:

$$L_{vehicle}=3.4\;m$$

Nevertheless, there is another way of obtaining the vehicle length by using the time during which the loop is fully occupied by the vehicle. In fact, if we call *L*_*loop*_ the total length of the loop, it will be satisfied that the time during which the entire loop is occupied (*t*_5_ − *t*_3_) will be equal to the difference between the vehicle size and the length of the loop divided by the vehicle speed, therefore, the vehicle length obtained by this method (*L*_vehicle*_) will be:

$$L_{vehicle*}=L_{loop}\;+\;V_{vehicle}\;(t_{5}\;-\;t_{3})=2\;+\;52.7\;\cdot (73\;-\;46)\;/\;1000=3,4\;m$$

Which gives the same value as the previous method. In addition, both methods match the exact length of the vehicle. Taking into account that we are working with measures of the order of meters, the result is very precise.

### Direction of traffic from magnetic profiles

The use of the double loops is capable of determining the speed and vehicle length, but it is also the ideal system to obtain the direction of traffic with the use of only a double loop. In fact, several previous figures showed the evolution of the oscillation frequency of the sensor system when the vehicle moved from the positive half-axis *X* towards the negative half-axis *X*, in which the positive section of the loop had a number of turns (*N*_1_) smaller than the negative section (*N*_1_ + *N*_2_).

With this configuration it can be verified that the evolution of the frequency deviation as a function of time starts with a reduced slope when the front of the vehicle enters the positive section, and then, an increase in slope occurs when the part front of the vehicle is penetrating into the negative section of the loop. If we consider what happens in the second part of the graph, it can be observed that, initially, when the rear part of the vehicle begins to leave the positive section of the loop, the descent slope is abrupt, but much less than when the rear part of the vehicle begins to leave the negative section of the loop, where an increase in the slope takes place.

**Fig 15:**
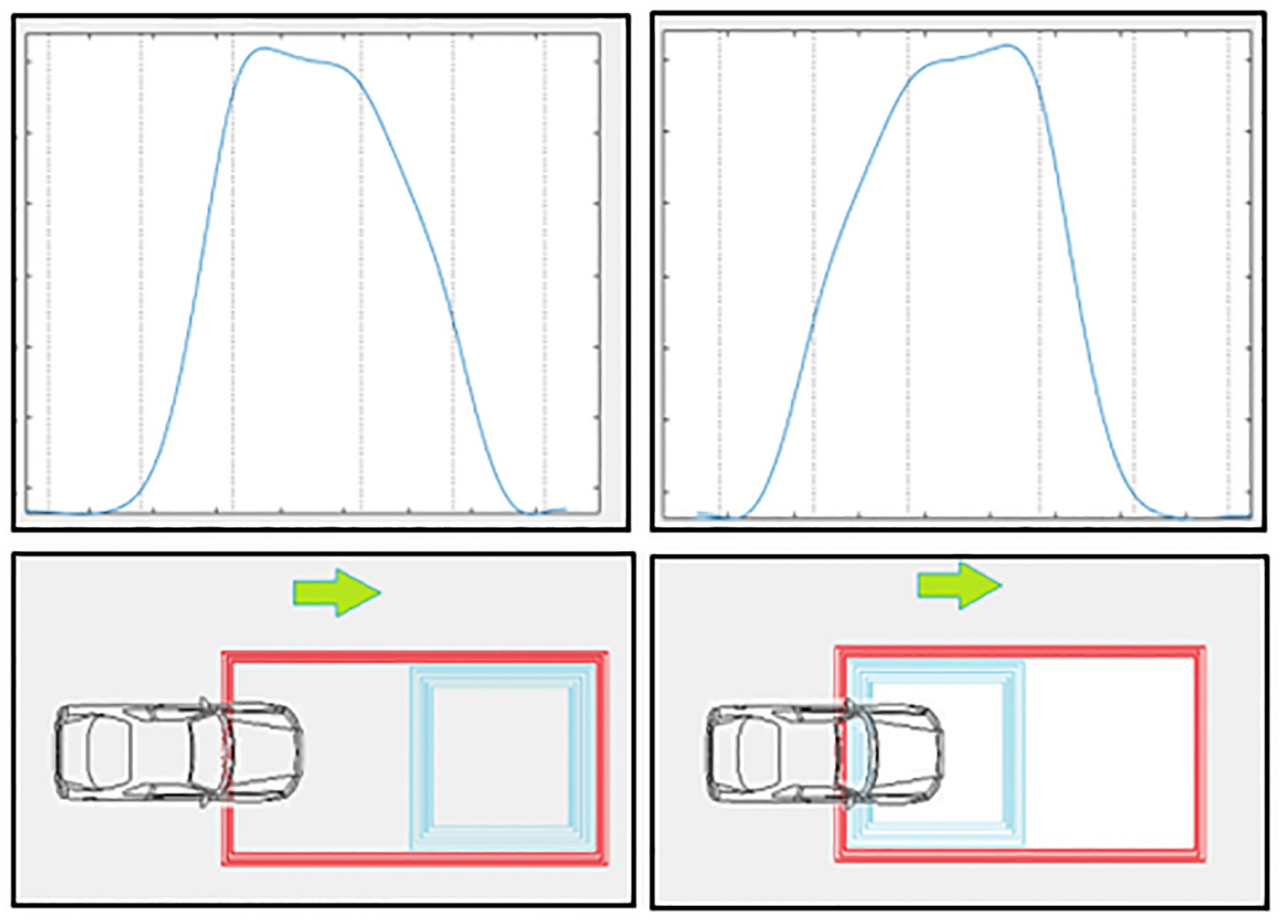
Different magnetic profiles according to the direction of traffic.

The reason for all this, as shown in Eq 10, is due to the fact that changes in the oscillation frequency are function of the square of the mutual inductance between the vehicle and the loop, and that mutual inductance is a function of the mutual flux concatenated between both, which depends on their relative position and the number of turns. Then, if we inversed the geometry of the loop or the direction of movement of the vehicle we would see that the evolution of the deviation of the frequency as a function of time would be reversed. We would start with a high rise slope, which would later decrease. Moreover, during the second half of the signal, it would be observed that the initial descent slope would be very high and then it will be decreasing. Therefore, for a given configuration of the double loop, it is enough to observe the evolution in the slope of the magnetic profile to know the direction of traffic. An example is shown in Fig 15.

## Conclusions

After the presentation of the double loop, where geometry, construction, operating mode and three possible ways to calculate its inductance was explained, this paper aimed to present the magnetic profiles generated by these loops and to demonstrate that the typical traffic parameters, previously calculated with two single loops per lane, can be obtained more easily with this new loop model.

The purpose of traffic engineering is to provide maximum efficiency and safety to users, thus minimizing the costs associated with traffic such as accidents, congestion, environmental impacts and economic costs. And for this, the basic fundamentals of study are traffic parameters such as intensity, speed, density, occupation or traffic direction, which today are still basically measured by magnetic loops due to its low cost and well-known technology.

Then, with this paper we have contributed to the Intelligent Transportation Systems (ITS) sector with the sophistication of this widely-known sensor. With its implementation, its cost and the complexity of the measurement system would be reduced and the functionalities and benefits would be even better than those provided by the single loops. In addition, all these data could be used to generate better algorithms for traffic management in cities. For that reason, the new magnetic profiles have been analyzed and it has explained how to calculate the different traffic parameters from these in a simple way.

## Supporting information

S1 File Data. This file contains two Excel documents. One of them shows the data recorded by the SCT-CEM-4 device for the three types of vehicles used and the other one shows the data referring to the extraction of parameters from the magnetic profiles.
(RAR)
